# Exploring the Diverse Functional and Regulatory Consequences of Alternative Splicing in Development and Disease

**DOI:** 10.3389/fgene.2021.775395

**Published:** 2021-11-24

**Authors:** M. Brandon Titus, Adeline W. Chang, Eugenia C. Olesnicky

**Affiliations:** University of Colorado Colorado Springs, Colorado Springs, CO, United States

**Keywords:** alternative splicing, RNA localization, splicing factors, RNA binding proteins, poison exons, premature termination codon (PTC), alternative last exon (ALE)

## Abstract

Alternative splicing is a fundamental mechanism of eukaryotic RNA regulation that increases the transcriptomic and proteomic complexity within an organism. Moreover, alternative splicing provides a framework for generating unique yet complex tissue- and cell type-specific gene expression profiles, despite using a limited number of genes. Recent efforts to understand the negative consequences of aberrant splicing have increased our understanding of developmental and neurodegenerative diseases such as spinal muscular atrophy, frontotemporal dementia and Parkinsonism linked to chromosome 17, myotonic dystrophy, and amyotrophic lateral sclerosis. Moreover, these studies have led to the development of innovative therapeutic treatments for diseases caused by aberrant splicing, also known as spliceopathies. Despite this, a paucity of information exists on the physiological roles and specific functions of distinct transcript spliceforms for a given gene. Here, we will highlight work that has specifically explored the distinct functions of protein-coding spliceforms during development. Moreover, we will discuss the use of alternative splicing of noncoding exons to regulate the stability and localization of RNA transcripts.

## Introduction

Alternative splicing is a fundamental mechanism that regulates eukaryotic gene expression and expands the number of possible transcript spliceforms and protein isoforms from a relatively small number of individual genes. In fact, alternative splicing is widespread among Metazoa, and it is estimated that approximately 95% of human genes exhibit alternative splicing (Wang et al., 2008). Through the process of choosing which coding exons will be maintained in a fully processed mRNA, alternative splicing has the potential to generate unique protein isoforms from a single gene that may have distinct functions or subcellular localizations from one another. However, the alternative splicing of noncoding exons, such as those of the 5′ and 3′ untranslated regions (UTRs), also generates unique spliceforms that may affect RNA export, RNA subcellular localization, stability, and translational efficacy.

To appreciate the potential that alternative splicing has for generating distinct functional gene products, it is important to recognize the extent to which alternative splicing is utilized in an organism’s life. Large transcriptomic datasets have demonstrated the differential expression of spliceforms in different tissues, developmental time points, and disease states. However, despite the vast data showing the use of alternative splicing, there is a relative paucity of examples for how different spliceforms from the same gene have different functions. In fact, one might argue that alternative splicing is the most enigmatic aspect of the regulation of gene expression (Tress et al., 2017; Rodriguez et al., 2020). This is likely due, in part, to the fact that it is often a significant technical challenge, even with sophisticated molecular tools, to discern the functions of different spliceforms of a gene. Nonetheless, it is important for the scientific community to tackle the Herculean task of elucidating spliceform-specific functions of genes (Ule and Blencowe, 2019). An important first step is to cultivate a greater awareness of the fact that different spliceforms may have different functions. Consequently, a nonsense mutation might be a null allele of one spliceform but may not impact another spliceform at all. Thus, taking care in interpreting experimental results in the context of spliceforms is essential. Furthermore, more spliceform specific mutant strains and antibodies should be generated to facilitate comprehensive studies of isoform functions. However, it is not until a concerted effort is made to generate such tools and perform such isoform-specific studies that the role alternative splicing plays in the cell will be fully appreciated.

In this review, we will first highlight the extensive use of alternative splicing to create unique expression patterns in different tissues, sexes, and stages during an animal’s life cycle. We will then discuss how alternative splicing can impart complex regulation of gene expression, facilitate the subcellular localization of RNAs, and review evidence for distinct functions among different spliceforms for select genes.

## Alternative Splicing Facilitates Tissue-, Age- and Sex-Specific Gene Expression

It is estimated that the human genome encodes over 1,500 RNA binding proteins (RBPs) (Gerstberger et al., 2014) that play diverse roles in RNA regulation including RNA stability, splicing, RNA localization, and other forms of RNA processing. Most RBPs are expressed within a broad range of tissues (Van Nostrand et al., 2020) and it is estimated that only 2–6% of RBPs show tissue-specific expression in humans. Nonetheless, there are tissue specific differences in the extent to which certain RNA regulatory processes, such as alternative splicing, are used. For example, both the nervous system and striated muscle have some of the highest levels of alternative splicing (Doxakis, 2014; Gerstberger et al., 2014). It is therefore not surprising that disruption of RBP function is implicated in cardiac and neurological disorders, such as amyotrophic lateral sclerosis, autism spectrum disorder, myotonic dystrophy, and cardiomyopathy (Baralle and Giudice, 2017).

One of the primary regulators of alternative splicing is a subset of RBPs identified as splicing factors. Two of the most common splicing factors are serine and arginine-rich (SR) proteins and heterogeneous nuclear ribonucleoproteins (hnRNPs) (Chen and Manley, 2009; Busch and Hertel, 2012; Fu and Ares, 2014). SR proteins are composed of two or more RNA recognition motifs and a serine-arginine rich domain that work in concert to recruit the core splicing machinery to bound splice sites, usually as a mechanism for exon inclusion (Chen and Manley, 2009; Busch and Hertel, 2012; Fu and Ares, 2014). Conversely, hnRNPs often work to block the recruitment of the core splicing machinery, resulting in exon exclusion (Chen and Manley, 2009; Busch and Hertel, 2012; Fu and Ares, 2014).

A recent survey of tissue-specific splicing showed that 95% of the tissue-specific splicing events that were supported through both proteomic and transcriptomic analyses were highly conserved and likely evolved prior to the ancestors of lobe-finned fish. However, it is important to note that the proteomic analyses utilized in this study may only detect the highest abundance proteins and may therefore not provide a comprehensive glimpse into the number of actual proteins that can be generated from the alternative splicing of a given gene. Nonetheless, the results of this study support previous findings that the brain and cardiac tissue have the highest levels of alternative splicing. In particular, the frontal cortex, fetal brain, spinal cord, and retina showed the most extensive alternative splicing. Furthermore, human coding genes are thought to have up to an average of ten different spliceforms, with approximately half of these spliceforms affecting the protein sequence (Rodriguez et al., 2020). Given the potential for alternative splicing to dramatically increase human proteomic complexity, there is a critical gap in our understanding of how different the functions are among distinct protein isoforms within a given tissue and between different tissues.

Alternative splicing also has the potential to create unique gene expression profiles between the sexes. A recent survey of gene expression across 44 human tissues in the different sexes showed that 37% of all genes have sex-biased expression in at least one tissue. However, this particular study did not examine alternative splicing signatures in these tissues or between the sexes (Oliva et al., 2020). This suggests that even more extensive sex-biased gene expression may exist in humans. Indeed, sex biased splicing has been observed in the brain, muscle and liver, and may contribute to sex differences in disease (Khramtsova et al., 2019). For example, many genes that exhibit sex biased splicing are associated with reproductive disorders or with diseases that have a sex bias in incidence (Deschênes and Chabot, 2017; Tower, 2017). Nonetheless, few splicing factors or spliceforms have been studied in detail with respect to their sex-specific functions.

In addition to tissue- and sex-specific or sex-biased alternative splicing, splicing patterns can also change during the life of an organism. In fact, changes in alternative splicing patterns and in splicing machinery are associated with senescence and aging (Deschênes and Chabot, 2017; Li et al., 2017; Wang et al., 2018). For example, there is a global increase in intron retention during aging in adult *Drosophila.* Yet distinct protein classes are differentially affected by this intron retention during different stages of aging, suggesting that intron retention may regulate specific aspects of the aging process. Importantly, these changes in intron retention are highly conserved in humans and mouse, as is the global increase in intron retention during aging (Adusumalli et al., 2019).

A comprehensive transcriptomic analysis of 48 different tissues from 544 human donors revealed approximately 50,000 splicing events that were correlated with age and were largely tissue specific. Importantly, changes in splicing were found in genes associated with the aging process, such as those involved in mitochondrial function, DNA repair and cell death. Moreover, substantial changes in the expression of components of splicing machinery were also detected as a function of age (Wang et al., 2018). Not surprisingly, defects in alternative splicing have also emerged as a common theme in aging-related diseases including vascular aging, accelerated aging (progeria), Alzheimer’s disease, amyotrophic lateral sclerosis, Frontotemporal lobar dementia, and myotonic dystrophy (Licatalosi and Darnell, 2006; Tazi et al., 2009; Anthony and Gallo, 2010; Lemmens et al., 2010; Strong, 2010; Mills and Janitz, 2012; Doxakis, 2014; Deschênes and Chabot, 2017). For example, intron retention is increased in brains of Alzheimer’s disease patients as compared to healthy controls (Adusumalli et al., 2019). Furthermore, the aggregation of the protein Tau, commonly associated with Alzheimer’s disease and other tauopathies, such as frontotemporal dementia with parkinsonism-17 (FTDP-17), has been shown to disrupt the localization of several known splicing factors (Jiang et al., 2021; Lester et al., 2021). Specifically, Tau oligomerization results in the mislocalization of the splicing regulators HNRNPA2B1 and SRRM2 from the nucleus to the cytoplasm (Jiang et al., 2021; Lester et al., 2021). Additionally, U1 and U2 spliceosomal RNAs localize to Tau aggregates. Furthermore, transcriptomic analysis of *tau* deficient cell lines revealed splicing defects in 226 genes, with intron retention representing approximately 50% of these defects and an additional 25% of these splicing defects being attributed to alternate usage of first or last exons (Lester et al., 2021). Thus, aberrant alternative splicing may represent a major molecular mechanism underlying the pathophysiology of many tau-mediated neurological disorders.

The levels of expression of individual isoforms can also provide an additional layer of tissue specific gene expression. Indeed, the ratio of isoform expression has been shown to be critical to normal cell function. For example, the alternative splicing of exon 10 of microtubule-associated protein Tau (MAPT) results in Tau proteins with either 3 (3R) or 4 (4R) repeats of a microtubule-binding domain. In healthy brain tissue there is a one-to-one ratio of 4R–3R isoforms. However, disruption of this ratio in either direction has been identified as a causal factor in variations of frontotemporal dementia (FTD) (Hong et al., 1998; Stanford et al., 2003).

While transcriptomic analyses have recently started to identify large scale changes in tissue-, sex- and age-specific splicing patterns, there remains a large gap in our understanding of how these alternative spliceforms contribute to the specification and function of distinct tissues, to the establishment of sex-specific differences in organisms and to the changes that occur during the process of aging.

## Alternative Splicing Coupled With Nonsense Mediated Decay

**FIGURE 1:**
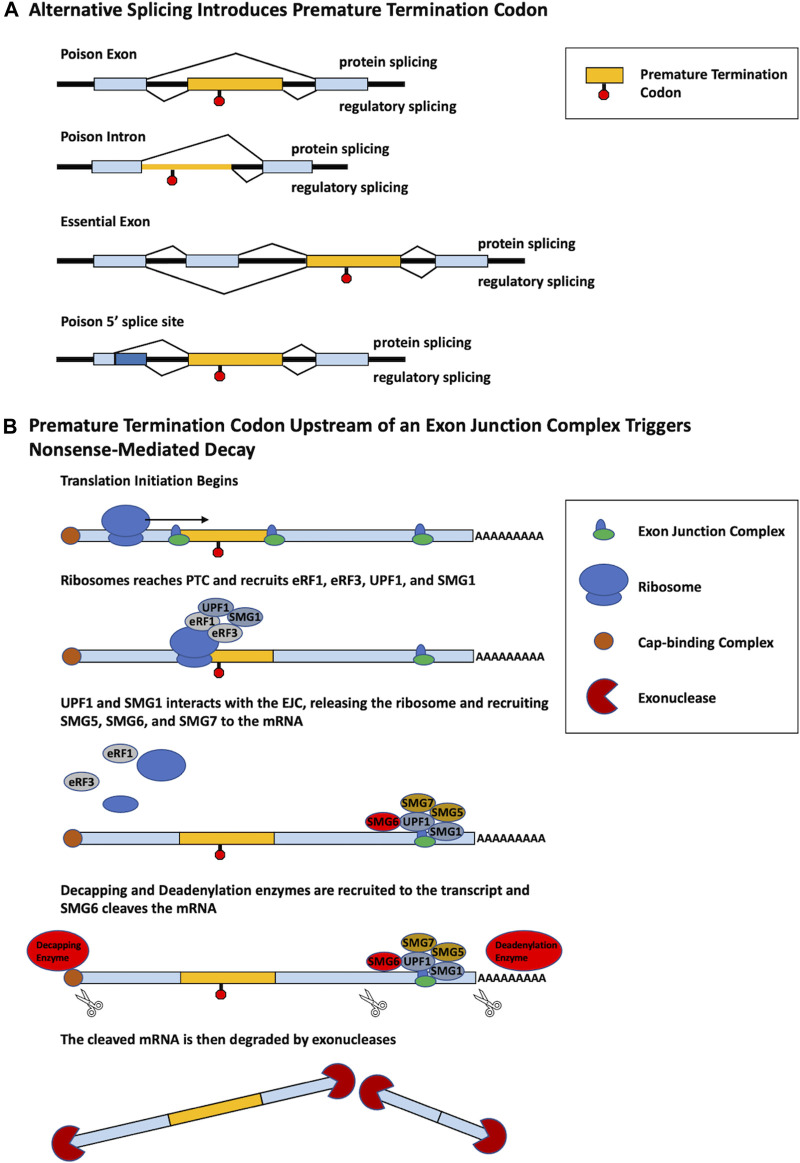
Different alternative splicing patterns can induce PTC inclusion that triggers NMD. **(A)** Multiple different alternative splicing events produce PTC-containing transcripts. Poison exons are cassette exons that contain an in-frame PTC and induce NMD when spliced into a transcript. The retention of a poison intron containing a PTC induces NMD. Essential exons induce NMD when skipped. An alternative poison 5′ splice site induces a frame shift that generates a PTC. For each model, splicing that generates a protein is shown on top, whereas regulatory splicing that results in AS-NMD is shown below. **(B)** PTC-containing mRNA is degraded by the process of NMD. Translation initiation begins to produce a polypeptide from the mRNA. Upon reaching the PTC, the proteins eRF1, eRF3, UPF1, and SMG1 are recruited to the ribosome. Recognition and interaction with a downstream EJC by UPF1 and SMG1 triggers the release of the ribosome and recruitment of SMG5, SMG6, and SMG7. Decapping and deadenylation enzymes are then recruited to the mRNA cleaving the 5′ cap and polyA tail, furthermore SMG6 cleaves the mRNA in two. The PTC-containing mRNA, no longer being protected, is then degraded by exonucleases.

While changes in alternative splicing are often thought to produce distinct functional protein isoforms, alternative splicing can also be used as a form of gene regulation. Alternative splicing coupled with nonsense-mediated decay (AS-NMD) is a mechanism of gene regulation in which alternative splicing within a pre-mRNA introduces a premature termination codon (PTC), generating transcripts targeted by nonsense-mediated decay (NMD) (Lewis et al., 2003). NMD is a critical RNA surveillance mechanism that promotes the degradation of mRNAs containing PTCs. The most common mechanism of NMD employs the use of the exon junction complex (EJC), which is a protein complex that forms at the junction of two exons in a pre-mRNA transcript. During the process of translation, the ribosome typically removes EJCs. However, when a PTC is located more than 50–55 nucleotides upstream of an EJC, the ribosome cannot remove the EJC. The PTC is bound by the proteins UPF1 and SMG1, which form a complex with eukaryotic release factors 1 and 3 (eRF1 and eRF3). This complex interacts with the EJC and ultimately recruits decapping and deadenylation enzymes which render the mRNA a target for 5′-3′ and 3′-5′ exonucleases (Figure 1; Kurosaki and Maquat, 2016; García-Moreno and Romão, 2020).

AS-NMD prevents the translation of a functional full-length protein, a process termed “regulated unproductive splicing and translation” (Lewis et al., 2003). PTCs may be introduced through usage of alternative exons containing in-frame PTCs, termed poison exons (PEs), or the skipping of essential exons which generate a downstream PTC. Additionally, PTCs can be generated through the retention of a poison intron (PI) or the use of an alternative 5′ splice site (Figure 1). PTCs may also be generated by alternative splicing in noncoding regions. For example, splicing factor SC35/serine and arginine rich splicing factor 2 (SRSF2) autoregulates its own expression through splicing events in the 3′UTR, generating an exon-exon junction downstream of the normal termination codon. The termination codon is then recognized as a PTC, targeting the transcript for NMD (Dreumont et al., 2009).

As PEs have just recently emerged as important components in regulating gene expression and maintaining homeostasis of cellular proteins, this review will report the current findings on the physiological importance of PE regulation.

## Extensive Use of AS-NMD Across Metazoa

PEs often overlap ultraconserved elements, which are segments of DNA that are greater than 200 base pairs in length and show 100% alignment between human, mouse, and rat. This suggests that PE regulation has highly conserved functional importance (Ni et al., 2007; Saltzman et al., 2008). This is underscored by reports that NMD-candidate isoforms account for approximately one third of known human alternative isoforms (Green et al., 2003; Lewis et al., 2003). Interestingly, genes containing PEs are enriched for those involved in RNA splicing (Ni et al., 2007; Thomas et al., 2020). Indeed, most human genes encoding SR proteins are regulated by the alternative splicing of ultraconserved elements that facilitate the introduction of PEs into transcripts. Many SR proteins bind to their own pre-mRNAs and promote their own “unproductive” splicing by PE inclusion to negatively autoregulate or maintain protein levels (Lareau et al., 2007; Lareau and Brenner, 2015; Leclair et al., 2020). It is interesting to note, however, that the PEs found in SR protein-encoding genes are different from one another, suggesting that RBPs may be under strong selective pressure to acquire, retain or replace existing PEs (Lareau and Brenner, 2015). Transcriptomic sequencing of both the murine and human brain cortex revealed over 1,000 PEs that are predicted to be subject to NMD. A significant proportion of these exons are found in transcripts encoding RBPs, with 84 different RBP-encoding genes harboring NMD sensitive exons. Nonetheless, PEs are also observed among 52 genes encoding chromatin regulators, though AS-NMD regulation of chromatin regulators likely arose in the mammalian lineage (Yan et al., 2015).

In a detailed study of alternative splicing across 309 human genes, Mudge et al. determined that AS-NMD is a widespread phenomenon across vertebrates. In fact, 22% of AS-NMD events for these 309 genes are conserved in non-mammalian vertebrates, often with sequence conservation. Yet interestingly, there is more than a three-fold increase in AS-NMD events in humans compared to mice within this dataset (Mudge et al., 2011). Since this particular study only focused on 1% of the human genome, more comprehensive studies involving the entire human genome are needed to fully determine if AS-NMD is generally used at higher rates in humans as compared to other vertebrate genomes. Moreover, while certain AS-NMD events may show sequence conservation within PEs, the use of PEs and AS-NMD as a regulatory mechanism may be conserved, but not at the sequence level. For example, the *RNA binding motif protein 39* (*RBM39*) gene, which encodes a splicing factor, has 42 annotated transcripts in humans and 24 transcript isoforms in mice. Six of these transcripts, two of which contain PEs, are conserved between mouse and human. While both of these PEs are highly conserved among mammals, one of these isoforms does not have any sequence similarity in non-mammalian vertebrate genomes (Mudge et al., 2011). Nonetheless, we have found that the *Drosophila* ortholog of *RBM39*, termed *caper,* also has multiple transcript isoforms, including three different poison exons. However, none of the PE sequences can be aligned to the mouse or human genome, suggesting that the mechanism, but not the sequence, of PE-mediated regulation of *caper/RBM39* expression is strongly selected for. Although, it should be noted that sequence conservation has been observed between mice and humans, with approximately 16% of non-productive exon inclusion events being conserved at the sequence level. For example, the mouse and human *SCN1A* genes share 96% conservation at the exon and flanking intronic regions (Lim et al., 2020).

To better characterize the extent to which AS-NMD is conserved as a mechanism for regulating gene expression, we sought to characterize the number of genes that generate PTC-containing isoforms. Previous studies have identified the conservation of AS-NMD events in humans and mice (Mudge et al., 2011; Lim et al., 2020). However, such studies have not been performed in invertebrates such as *Drosophila* and *C. elegans*. The *Drosophila* genomic database, FlyBase, has over 692 genes that have at least one transcript with an annotation supporting the inclusion of a PTC (Larkin et al., 2021). However, our manual curation of the annotated transcripts included in FlyBase did not demonstrate clear indication of PTC containing variants for some of these genes. One explanation is that not all transcripts, especially low-frequency exon junctions, may be included in FlyBase (Larkin et al., 2021). To provide a conservative analysis of genes containing alternatively spliced PTCs, we manually screened the list of 692 genes for readily observable isoforms containing PTCs. In this analysis, PTCs were confirmed if inclusion of an alternative exon or 5′ splice site resulted in disruption of translation of a downstream exonic region that was protein coding in another transcript. Using this method, we were able to verify 374 genes with transcripts that contain PTCs through alternative splicing (Supplementary Table S1). Moreover, using previous datasets that identified genes that either contained PTCs or were regulated by the NMD pathway in humans (Wittmann et al., 2006; Mudge et al., 2011; Schmidt et al., 2015; Yan et al., 2015; Lim et al., 2020), in mouse (Mcilwain et al., 2010; Mudge et al., 2011; Weischenfeldt et al., 2012; Yan et al., 2015), and *C. elegans* (Ramani et al., 2009; Muir et al., 2018), we confirmed the conservation of AS-NMD events in a subset of genes.

To better understand the functional role of these genes, we performed GO Term analysis of the molecular functions for the list of genes using the ClueGO plugin for Cytoscape (Bindea et al., 2009). The number of enriched GO terms identified with a Benjamini-Hochberg corrected *p*-value of 0.05 or less for each species varied with humans having 60 enriched GO terms, mouse having 56, *Drosophila* with 27, and *C. elegans* having 7. Interestingly, the enrichment of the GO Term for RNA binding is amongst the highest by number of genes in each species (Tables 1–4). This is in alignment with the high prevalence of PTC enabled nonsense-mediated decay of RBPs and splicing factors that has previously been reported in human and mouse. Furthermore, we identified nine genes, all of which encode splicing factors, that are conserved AS-NMD targets across all four species (Table 5). This is likely an underrepresentation of actual AS-NMD events as the depth of transcriptomic information regarding PTC-containing or NMD-sensitive genes is not equivalent between species. Nonetheless, this data demonstrates that the mechanism of AS-NMD is conserved across the Metazoa, and has a strong bias for being utilized in RBP encoding genes and, more specifically, splicing factors.

**TABLE 1:** GO term analysis for AS-NMD regulated genes in humans.

GOTerm	Adjusted *p*-value	Number of genes
Metal ion binding	0.0002	1,328
Nucleotide binding	0.0000	766
Ribonucleotide binding	0.0000	675
Purine nucleotide binding	0.0000	670
Purine ribonucleotide binding	0.0000	668
Purine ribonucleoside triphosphate binding	0.0000	640
RNA binding	0.0000	584
Adenyl nucleotide binding	0.0000	558
Adenyl ribonucleotide binding	0.0000	556
ATP binding	0.0000	534
Hydrolase activity, acting on acid anhydrides, in phosphorus-containing anhydrides	0.0000	493
Pyrophosphatase activity	0.0000	490
Nucleoside-triphosphatase activity	0.0000	471
ATPase activity	0.0000	203
GTPase binding	0.0019	197
Nucleoside-triphosphatase regulator activity	0.0014	130
mRNA binding	0.0000	121
GTPase regulator activity	0.0014	117
GTPase activator activity	0.0014	104
Methyltransferase activity	0.0000	94
Nuclease activity	0.0026	90
Helicase activity	0.0000	84
Nucleotidyltransferase activity	0.0022	76
S-adenosylmethionine-dependent methyltransferase activity	0.0000	71
DNA-dependent ATPase activity	0.0000	57
Single-stranded DNA binding	0.0008	54
Exopeptidase activity	0.0022	51
N-methyltransferase activity	0.0000	47
Exonuclease activity	0.0026	46
DNA helicase activity	0.0000	45
Single-stranded RNA binding	0.0001	43
Hydrolase activity, acting on carbon-nitrogen (but not peptide) bonds, in linear amides	0.0018	41
RNA helicase activity	0.0000	41
Protein methyltransferase activity	0.0000	39
3′-5′ exonuclease activity	0.0026	32
Exonuclease activity, active with either ribo- or deoxyribonucleic acids and producing 5′-phosphomonoesters	0.0026	31
NAD binding	0.0014	30
Aminopeptidase activity	0.0022	25
Polyubiquitin modification-dependent protein binding	0.0037	24
2-oxoglutarate-dependent dioxygenase activity	0.0026	23
Aminoacyl-tRNA ligase activity	0.0026	22
5′-3′ RNA polymerase activity	0.0022	22
Exoribonuclease activity	0.0026	22
Pre-mRNA binding	0.0019	20
Ran GTPase binding	0.0019	20
Positive regulation of telomerase activity	0.0026	19
3′-5′-exoribonuclease activity	0.0026	19
Mannosyltransferase activity	0.0007	18
NF-kappaB binding	0.0025	17
Poly-pyrimidine tract binding	0.0001	16
Poly-purine tract binding	0.0001	16
Telomerase RNA binding	0.0015	15
Phosphotransferase activity, for other substituted phosphate groups	0.0015	15
Prenyltransferase activity	0.0015	13
Poly(A) binding	0.0001	13
Mismatched DNA binding	0.0026	10
Acyl-CoA dehydrogenase activity	0.0027	8
Protein-arginine N-methyltransferase activity	0.0027	8
Protein prenyltransferase activity	0.0015	7
Protein-arginine omega-N symmetric methyltransferase activity	0.0027	5

**TABLE 2:** GO term analysis for AS-NMD regulated genes in mouse.

GOTerm	Adjusted *p*-value	Number of genes
RNA binding	0.0000	104
mRNA binding	0.0000	38
Ubiquitin protein ligase binding	0.0003	19
Pre-mRNA binding	0.0000	14
mRNA 3′-UTR binding	0.0000	14
RNA polymerase II-specific DNA-binding transcription factor binding	0.0175	13
Active ion transmembrane transporter activity	0.0078	13
Active ion transmembrane transporter activity	0.0022	13
Proton transmembrane transporter activity	0.0022	11
Single-stranded DNA binding	0.0000	11
Hormone receptor binding	0.0175	10
Single-stranded RNA binding	0.0002	10
Nuclease activity	0.0310	10
Nucleotidyltransferase activity	0.0215	9
rRNA binding	0.0008	8
Helicase activity	0.0284	8
Ubiquitin-like protein-specific protease activity	0.0153	7
Translation factor activity, RNA binding	0.0087	7
Nuclear receptor binding	0.0175	7
Ribonuclease activity	0.0310	7
Antiporter activity	0.0078	6
Antiporter activity	0.0022	6
ATPase-coupled cation transmembrane transporter activity	0.0022	6
Regulatory RNA binding	0.0000	6
tRNA binding	0.0168	5
RNA helicase activity	0.0284	5
Poly-pyrimidine tract binding	0.0002	5
Poly-purine tract binding	0.0002	5
3′-5′ exonuclease activity	0.0310	5
Exonuclease activity, active with either ribo- or deoxyribonucleic acids and producing 5′-phosphomonoesters	0.0310	5
Telomeric DNA binding	0.0000	5
Phosphatidylinositol-3-phosphate binding	0.0208	4
Ionotropic glutamate receptor binding	0.0160	4
Ubiquitin-like protein conjugating enzyme activity	0.0195	4
snRNA binding	0.0164	4
Translation elongation factor activity	0.0087	4
Pre-mRNA 3′-splice site binding	0.0000	4
Ion transmembrane transporter activity, phosphorylative mechanism	0.0022	4
Cholesterol transfer activity	0.0159	3
Hexosaminidase activity	0.0105	3
Intramolecular oxidoreductase activity, interconverting aldoses and ketoses	0.0023	3
5S rRNA binding	0.0008	3
Pre-mRNA intronic binding	0.0004	3
mRNA CDS binding	0.0004	3
Anion:anion antiporter activity	0.0078	3
Sequence-specific single stranded DNA binding	0.0000	3
Large ribosomal subunit rRNA binding	0.0205	2
5′-nucleotidase activity	0.0281	2
Uridylyltransferase activity	0.0156	2
C-rich single-stranded DNA binding	0.0034	2
Poly(G) binding	0.0002	2
Voltage-gated chloride channel activity	0.0078	2
Regulatory region RNA binding	0.0000	2
N6-methyladenosine-containing RNA binding	0.0000	2
Positive regulation of telomerase RNA reverse transcriptase activity	0.0000	2
G-rich strand telomeric DNA binding	0.0000	2

**TABLE 3:** GO term analysis for AS-NMD regulated genes in *Drosophila*.

GOTerm	Adjusted *p*-value	Number of genes
Metal ion binding	0.0022	74
Nucleotide binding	0.0076	52
RNA binding	0.0022	36
Poly(A) RNA binding	0.0022	22
mRNA binding	0.0022	19
Substrate-specific channel activity	0.0078	14
Ion channel activity	0.0078	14
Calcium ion binding	0.0096	13
Metal ion transmembrane transporter activity	0.0078	13
Gated channel activity	0.0078	12
Actin binding	0.0051	11
Cation channel activity	0.0078	10
Kinase binding	0.0092	6
Divalent inorganic cation transmembrane transporter activity	0.0078	6
Adenylate cyclase activity	0.0024	5
Glutamate receptor activity	0.0048	5
Calcium ion transmembrane transporter activity	0.0078	5
Pre-mRNA binding	0.0029	4
Calcium-dependent phospholipid binding	0.0101	3
Translation regulator activity, nucleic acid binding	0.0079	3
Peptidase activator activity	0.0035	3
Intracellular ligand-gated ion channel activity	0.0078	3
Tau-protein kinase activity	0.0050	2
Voltage-gated ion channel activity involved in regulation of postsynaptic membrane potential	0.0050	2
Voltage-gated sodium channel activity	0.0050	2
Cysteine-type endopeptidase activator activity involved in apoptotic process	0.0035	2
Peptidase activator activity involved in apoptotic process	0.0035	2
Cholesterol binding	0.0048	2
Glutamate binding	0.0048	2
Ligand-gated calcium channel activity	0.0078	2
Calcium-release channel activity	0.0078	2
Calcium: cation antiporter activity	0.0078	2
Store-operated calcium channel activity	0.0078	2

**TABLE 4:** GO Term Analysis for AS-NMD Regulated Genes in *C. elegans*.

GOTerm	Adjusted *p*-value	Number of genes
RNA binding	0.0002	39
Single-stranded RNA binding	0.0055	5
Neuropeptide hormone activity	0.0067	4
Neuropeptide receptor binding	0.0067	4
Oxidoreductase activity, acting on the CH-NH_2_ group of donors, oxygen as acceptor	0.0024	3
D-aspartate oxidase activity	0.0024	2
D-glutamate oxidase activity	0.0024	2

**TABLE 5:** AS-NMD regulated genes conserved across the Metazoa.

Human	Mouse ortholog	Drosophila ortholog	*C. elegans* ortholog
*RBM39*	*Rbm39*	*Caper*	*rbm-39*
*RSRC2*	*Rsrc2*	*CG6340*	*ZC262.2*
*SF3B3*	*Sf3b3*	*Sf3b3*	*teg-4*
*SFSWAP*	*Sfswap*	*su(w[a])*	*swp-1*
*SRRM1*	*Srrm1*	*corn*	*rsr-1*
*SRRT*	*Srrt*	*Ars2*	*E01A2.2*
*SRSF3*	*Srsf3*	*Rbp1*	*rsp-6*
*SRSF4*	*Srsf4*	*B52*	*rsp-1*
*TRA2A*	*Tra2a*	*Tra2*	*rsp-8*

## PE Usage as an Autoregulatory Mechanism for the Expression of SR Proteins and hnRNPs

AS-NMD represents a common mechanism for the autoregulation of splicing factors and may be a critical mechanism for regulating the expression levels of distinct spliceforms. Indeed, transient high levels of the SR protein serine and arginine rich splicing factor 7 (SRSF7) in wild type mouse cells induce the binding of SRSF7 to its own transcripts and promote the inclusion of its PE, reducing levels of functional SRSF7 protein (Königs et al., 2020). Surprisingly, prolonged overexpression of SRSF7 mRNA in P19 mouse cells not only promotes PE inclusion but also protects the PE-containing transcript from NMD, resulting in the translation of two distinct truncated proteins termed Split-ORFs, one of which effectively competes with full-length SRSF7 for binding the SRSF7 transcript (Königs et al., 2020). The binding of these short proteins results in an elongated transcript that excludes the PE but retains introns 3a, 3b, and 5 (Königs et al., 2020). These intron-containing transcripts provide binding sites that promote SRSF7 binding and oligomerization, forming nuclear bodies which may sequester different SRSF7 mRNA isoforms. The sequestration of these transcripts within the nucleus ultimately reduces the levels of translatable transcripts within the cytoplasm, as well as functional SRSF7 within the nucleus, thereby restoring normal SRSF7 protein levels (Königs et al., 2020). Other genes are also predicted to generate Split-ORFs, and these genes are enriched for those encoding RBPs including other SR proteins, such as heterogeneous nuclear ribonucleoprotein L (hnRNPL) and RBM39 (Königs et al., 2020).

The alternative splicing of PEs within other SR proteins is also regulated by an extensive autoregulatory and cross-regulatory network (Leclair et al., 2020). In an iCLIP analysis of serine and arginine rich splicing factor 3 (SRSF3) binding patterns in mouse P19 cells, SRSF3 was shown to not only autoregulate its own expression through the splicing of poison exons but was also found to cross-regulate PE usage of other SR protein-encoding genes such as *SRSF2, serine and arginine rich splicing factor 5* (*SRSF5*), and *SRSF7* (Änkö et al., 2012). Transcriptomic analyses of induced pluripotent stem cells differentiated into various cell types including neuronal, pancreatic and lung epithelial cells indicate that PEs of SR protein-encoding transcripts are increasingly included as cells differentiate but show low inclusion within undifferentiated cells. Moreover, analyses of thirteen different human tumor expression profiles as compared to noncancerous control tissues from the Cancer Genome Atlas indicate dysregulated alternative splicing of PE-containing transcripts, suggesting that PE splicing of SR protein-encoding transcripts may be a potential therapeutic target (Leclair et al., 2020). Similarly, Thomas et al. found over 500 highly conserved PEs in HeLa and PC9 lung adenocarcinoma cells, many of which are essential for cell growth and fitness, suggestive of their roles in cancer progression (Thomas et al., 2020). The study also identified PEs that exhibit tumor-suppressor activity and revealed a link between the skipping of tumor-suppressive PE and poor disease prognosis in adenocarcinoma patients (Thomas et al., 2020).

The complexity of PE regulation is again highlighted by its regulation of the SR protein transformer 2 beta (Tra2β), an oncogene frequently overexpressed in various cancers. Cellular Tra2β concentration is autoregulated by the binding of full length Tra2β protein to exon splicing enhancer regions in a PE within exon two of the Tra2β transcript, promoting PE inclusion (Stoilov et al., 2004). Additionally, phosphorylation of Tra2β negatively regulates its binding to the Tra2β transcript and consequently PE inclusion (Stoilov et al., 2004). Tra2β PE is also cross-regulated by serine and arginine rich splicing factor 1 (SRSF1), SRSF3, and serine and arginine rich splicing factor 4 (SRSF4), SR proteins that decrease PE inclusion and compete with positive regulators, transformer 2 alpha (Tra2α) and Tra2β*,* to regulate PE usage (Leclair et al., 2020).

hnRNPs also autoregulate and cross-regulate one another by modulating PE usage (Ni et al., 2007; Saltzman et al., 2008; Rossbach et al., 2009). Intron 6 of the human HNRNPL pre-mRNA contains a highly conserved PE (exon 6A) whose inclusion is activated by HNRNPL binding. In a similar way, HNRNPL cross-regulates the expression of its paralog heterogeneous nuclear ribonucleoprotein L like (HNRNPLL) (Rossbach et al., 2009). Additionally, the presence of hnRNP binding motifs surrounding PEs in SR protein encoding transcripts indicate that hnRNPs can regulate PE inclusion and consequently the expression of SR proteins (Leclair et al., 2020). hnRNPs can also regulate the inclusion of other RBPs that are not classified as either SR or hnRNP proteins. For example, the RNA DExH-box helicase 9 (*DHX9*) is associated with Ewing sarcoma, an aggressive cancer of the bone and soft tissue that typically affects younger adults. Higher *DHX9* expression levels are correlated with worse prognosis for Ewing sarcoma patients, making *DXH9* a potential cancer therapeutic target. Moreover, inclusion of a poison exon, encoded by exon 6A, within the *DHX9* transcript results in a PTC which triggers *DXH9* mRNA for NMD*.* Using an siRNA screening approach, Palombo et al. identified heterogeneous nuclear ribonucleoprotein M (HNRNPM) and SRSF3 as negative regulators of exon 6A inclusion. Importantly, knockdown of *HNRNPM* or *SRSF3* lowered *DHX9* protein levels, decreased the viability and proliferation of an Ewing sarcoma cell line, and rendered the cells more sensitive to chemotherapeutics. Taken together, regulation of poison exon inclusion within the *DXH9* transcript may be a powerful target for treatment of Ewing sarcoma (Palombo et al., 2020).

## Aberrant PE Regulation is Implicated in Neurological Disorders

Given that RBP dysfunction and aberrant splicing are increasingly associated with myriad neurological disorders and that PEs are critical for the regulation of RBP expression, it is not surprising that aberrant PE regulation is implicated in neurological disorders. For example, G-patch domain containing 8 (GPATCH8) is an RBP that interacts with splicing factors in nuclear speckles and is likely involved in pre-mRNA processing. *Gpatch8* contains two PEs flanked by RBFOX sites, suggesting that PE inclusion is regulated by the RBP RBFOX (Chapman et al., 2019). Notably, *Gpatch8* is the murine ortholog of human zinc finger protein 804A (ZNF804A), a nuclear protein whose dysfunction is linked to schizophrenia (Chapman et al., 2019). Moreover, RNA binding fox-1 homolog 1 (RBFOX1) has also been implicated in schizophrenia in a large genome wide association study, suggesting yet another link between PEs and neurological disorders (Pardiñas et al., 2018). Rbfox proteins also regulate PE inclusion in genes associated with neurodevelopment. Vaquero-Garcia et al. detected a previously uncharacterized brain-specific PE, exon 14, in the murine *polypyrimidine tract binding protein 1* (*Ptbp1*) gene, which encodes a splicing factor involved in neurodevelopment. The PE is conserved in both mice and humans as well as other mammals, indicative of its functional importance. In mouse cortices, exon 14 is excluded from embryonic to P15 stages and included from P15 to adulthood, indicating that exon 14 inclusion is developmentally regulated. The authors also detected Rbfox binding sites downstream of exon 14 of Ptbp1 in both mouse and humans, suggesting that exon 14 inclusion is regulated by Rbfox. Indeed, nestin-specific *Rbfox1* knockout in one-month old mice resulted in a considerable decrease in exon 14 inclusion (Vaquero-Garcia et al., 2016).

Ptbp1 itself regulates PE usage during neurogenesis. Ptbp1 suppresses the inclusion of a highly conserved mammalian PE (exonN) in the gene encoding the cytoskeleton protein filamin, alpha (Flna) (Zhang et al., 2016). *Flna* exonN inclusion is developmentally regulated such that it is included in adult brain tissue but excluded in neural progenitor cells (NPCs). Additionally, conditional knockout of *Ptbp1* in the mouse cerebral cortex results in the increase of *Flna* exonN usage and premature differentiation of NPCs (Zhang et al., 2016). Thus, Ptbp1 regulation of *Flna* exonN usage may regulate NPC differentiation and cerebral cortex development. Importantly, *Flna* heterozygous null mutations in female humans are known to cause neuronal migration defects in the ventricular zone, which lead to brain malformations termed periventricular heterotopia (PVNH). Moreover, a rare intronic mutation in the *Flna* gene that causes inappropriate inclusion of exonN results in a more mild form of PVNH, further exemplifying the neurological consequences of aberrant alternative splicing (Zhang et al., 2016).

NOVA is a brain specific RBP that regulates alternative splicing and mRNA transport. Using cross-linking followed by immunoprecipitation and sequencing, Eom et al. (2013) identified nuclear and cytoplasmic NOVA RNA targets. GO term analysis shows that NOVA-regulated transcripts were enriched for genes with synaptic functions. Interestingly, while cytoplasmic NOVA binds its target RNAs predominantly near the 3′UTR, nuclear NOVA tends to bind its targets within introns. Moreover, nuclear NOVA regulates the alternative splicing of NMD sensitive isoforms for a number of its target RNAs. For example, NOVA suppresses the inclusion of PTC containing exon E15 in the *discs large MAGUK scaffold protein 3* (*Dlg3*) transcript in the mouse brain. Indeed, NOVA knockout mice have a marked decrease in both the protein coding *Dlg3* mRNA isoforms and DLG3 protein levels compared to controls. These NOVA knockout mice express PTC-containing *Dlg3* spliceforms that are not detected in controls. By contrast, NOVA also promotes the inclusion of a PTC containing exon (E17a) in the sodium channel, voltage-gated, type IX, alpha (*Scn9a*) transcript, leading to NMD of the transcript (Eom et al., 2013). Further analysis revealed that NOVA also mediates PE inclusion in *syntaxin 2* (*Stx2*) and prevents PE inclusion in *RAS protein-specific guanine nucleotide-releasing factor 1* (*Rasgrf1*) and *solute carrier family 4 (anion exchanger), member 3* (*Slc4a3*) transcripts, suggesting that NOVA regulation of PE inclusion is target dependent. Interestingly, the splicing of NOVA-regulated transcripts is perturbed by seizure activity, raising the possibility that NOVA function can be modulated through electrical activity. Heterozygous NOVA mice displayed spontaneous seizures and epilepsy, and seizures also induced shifts in the subcellular localization of NOVA from nucleus to cytoplasm. Therefore, it is possible that variations in electrical activity elicit changes in NOVA subcellular localization, and consequently, splicing of PE containing transcripts associated with epilepsy (Eom et al., 2013).

Interestingly, the incidence of PEs in seizure associated genes is not limited to NOVA. Indeed, of the ten genes that are most commonly associated with epilepsy, five have NMD sensitive exons including multiple that encode sodium channels (Aziz et al., 2021). Indeed, a recent study of developmental and epileptic encephalopathies patients uncovered five pathogenic variants of the *sodium voltage-gated channel alpha subunit 1* (*SCN1A*) gene, which encodes a type I voltage-gated sodium channel. Importantly, the pathogenic variants, all of which have intronic lesions, promote PE (exon 20N) inclusion leading to NMD of the SCN1A transcripts, thereby lowering expression levels of the sodium channel (Carvill et al., 2018). In a different study, CRISPR/Cas9 was used to knock-in (KI) a pathogenic, PE promoting, variant of *sodium channel, voltage-gated, type I, alpha* (*Scn1a*) to generate a Dravet Syndrome (DS) mouse model (Voskobiynyk et al., 2021). *Scn1a* +/KI mice exhibited decreased levels of *Scn1a* mRNA and protein in the brain, as well as an increase in exon 20N inclusion compared to control mice. Moreover, truncated proteins were not detected, indicating that the transcripts likely were subjected to NMD (Voskobiynyk et al., 2021). *Scn1a* +/KI mice display phenotypes previously reported in other DS models such as hyperactivity (increase in jumping and distance travelled), premature mortality, and spontaneous seizures (Voskobiynyk et al., 2021). This not only supports the use of *Scn1a* +/KI mice as a DS model, but specifically implicates PE (exon 20N) inclusion in DS pathogenesis. An analysis of RNA-seq data of wild type mouse cortex revealed that throughout development, the inclusion of exon 20N decreases, accompanied by the increase in overall *Scn1a* mRNA levels. A similar pattern was observed for *sodium channel, voltage-gated, type VIII, alpha* (*Scn8a*) (Voskobiynyk et al., 2021). *Scn8a* pre-mRNA contains two alternatively spliced exons, 18A and 18N. Exon 18N is a PE that is conserved in humans, mice, and fish (*Fugu rubripes*). Throughout the course of murine fetal brain development, transcripts containing exon 18N decrease while those containing 18A increase (Plummer et al., 1997). Additionally, analysis of cDNA from adult mouse tissues revealed that 18A containing *Scn8a* transcripts predominate in brain and spinal cord tissues while transcripts containing 18N are more abundant in non-neuronal tissues, indicating that the inclusion of exon 18N is regulated in a tissue-specific manner (Plummer et al., 1997). Thus, poison exon usage in *Scn8a* is regulated spatiotemporally to coordinate developmental neurogenesis in the mouse.

While we have primarily focused on the role of PEs in neurodevelopment and neurological disease, PE usage is also implicated in dystrophinopathies, which are characterized by progressive degeneration of skeletal muscle due to genetic lesions within the dystrophin encoding gene, *DMD*. *DMD* is one of the largest genes within the human genome, containing 79 protein-coding exons and 42 putative PEs. Importantly, an intronic pathogenic variant of the *DMD* gene may activate the inclusion of PEs, resulting in disease manifestation (Xie et al., 2020). While PE usage is implicated in dystrophinopathies, the pathophysiology of both myotonic dystrophy type 1 (DM1) and type 2 (DM2) is also associated with the sequestration of Muscleblind-like (MBNL) proteins to RNAs containing CUG or CCUG repeats (Sznajder and Swanson, 2019). This is further supported by the competition of RBPs MBNL1 and rbFox1 in binding the CCUG repeats, which is believed to be a reason for the milder phenotypes observed in DM2 compared to DM1. Additionally, increased expression of RBFox1 has been shown to correct splicing alterations and alleviate muscle atrophy in DM2 presenting tissues and organisms (Sellier et al., 2018). Thus, aberrant splicing is implicated in multiple different ways in dystrophinopathies.

## Targeting of AS-NMD Pathways in the Development of Therapeutics

Given that aberrant PE regulation is associated with myriad diseases, the development of therapeutics specifically targeting PE containing transcripts may provide an effective strategy for treating disease. One of the more recent breakthroughs in therapeutic treatments for aberrant PE regulation is a technique called targeted augmentation of nuclear gene output (TANGO). TANGO utilizes antisense oligonucleotides (ASOs) to bind to mRNA splice sites to manipulate splicing events in target genes. The use of ASOs to manipulate transcript splicing events has been demonstrated to be effective, in a dose-dependent manner, in decreasing the expression of PTC-containing transcripts, increasing the expression of functional transcripts, and increasing protein levels of targeted genes in cell culture. Furthermore, ASOs have been demonstrated to be effective when targeting PTC inclusion from multiple splicing events including cassette exons, alternative splice sites, and poison introns (Lim et al., 2020).

TANGO has also shown promise in the treatment of disease. For example, Dravet Syndrome (DS) is one of the most common developmental and epileptic encephalopathies affecting an estimate of 20,000 individuals in the United States. DS is characterized by seizures, cognitive regression, ataxia, and increased mortality. Furthermore, individuals with DS are at high risk of sudden unexpected death in epilepsy (SUDEP). Greater than 80% of DS cases are due to mutations in the *sodium voltage-gated alpha subunit 1* (*SCN1A*) gene, which is a subunit of the sodium channel NA_v_1.1 (Han et al., 2020; Helbig and Goldberg, 2021). Furthermore, the mutation of *SCN1A* that is associated with DS is characterized by a 5-fold increase in expression of a PE, which results in a 50% reduction of *Scn1a* mRNA and NA_v_1.1 protein levels (Voskobiynyk et al., 2021). However, treatment with ASOs targeting the *SCN1A* (ASO-22) has been demonstrated to be effective in reducing expression of the PE containing transcript, increasing the functional transcript, and increasing NA_v_1.1 protein expression in human neural progenitor cells. Furthermore, ASO-22 treatment has also been shown to have promising effects *in vivo*. Intracerebroventricular injections in the brains of P2 WT mice resulted in an observable decrease of the PTC-containing *Scn1a* transcripts and an increase in the functional *Scn1a* transcripts up to 30 days after injection. Additionally, injection of ASO-22 in a DS mouse model increased longevity nearly 4-fold and reduced the incidence of tonic-clonic seizures and SUDEP while showing no significant lethality in WT mice treated with ASO-22 (Han et al., 2020).

TANGO has also been demonstrated to be effective in treating several cancers that are impacted by AS-NMD. For example, the gene encoding the splicing factor SF3B1 is commonly mutated in cancer in a manner that results in the usage of abnormal 3′ splice sites. One of the mis-spliced genes identified in multiple *SF3B1*-mutated cancerous cell lines including erythroleukemic (K562) and uveal melanoma (UVM) is *bromodomain containing 9* (*BRD9*)*,* a component of the non-canonical BAF (ncBAF) chromatin remodeling complex. The role of *BRD9* in cancer was confirmed when knockout of *BRD9* conferred a growth advantage to UVM, cutaneous melanoma, and pancreatic cancer cells. Knockdown of *BRD9* also resulted in tumor growth, augmented melanocyte pigmentation, and decreased expression of melanocyte lineage specific genes in Melan-a cells. Additionally, knockdown of *BRD9* increased metastasis in mouse xenografts of murine melanoma or human UVM cells. Importantly, mutation in the *SF3B1* gene often results in the introduction of a PE. Inclusion of the PE triggers NMD, as indicated by the reduced half-life of *BRD9* mRNA and decreased expression of BRD9 protein in cell culture. Excitingly, ASOs designed to block the inclusion of the *BRD9* poison exon was sufficient to rescue mis-splicing, increase protein levels, and suppress tumor growth in both UVM cells and mice xenografted with UVM cells. The *BRD9-*targeting ASO was also shown to be effective in tumor suppression in rectal melanoma patient-derived xenografts (Inoue et al., 2019).

Decreased *Tra2β* PE inclusion is also seen in multiple different types of tumors, including breast cancer. However, the use of splice-switching ASOs targeting an ultraconserved region of the Tra2β transcript promotes Tra2β PE inclusion and thereby decreases Tra2β protein levels. Treatment of breast cancer cell lines with these splice-switching ASOs reduces their proliferation and viability, underscoring the potential of targeting PE-containing transcripts as an effective cancer therapeutic strategy (Leclair et al., 2020). Taken together, TANGO is a promising therapeutic technique in treating diseases associated with AS-NMD. Furthermore, the specificity and diversity of ASO targeting allows this technique to be extended to other AS-NMD associated diseases and to different types of mutations that specifically affect PE inclusion.

## RNA Localization

In addition to alternative splicing regulating the stability of transcripts through AS-NMD, the use of alternative exons can also regulate the localization of RNA transcripts within a cell. Subcellular localization of RNA, proteins and protein complexes is critical for proper cellular function, as is evidenced by the fact that improper localization of RNAs and proteins can result in disease (Hung and Link, 2011; Engel et al., 2020; Abouward and Schiavo, 2021; Fernandopulle et al., 2021). While localization of proteins can occur post-translationally, mRNAs are often localized to subcellular compartments and remain translationally quiescent during transport. However, upon reaching their final destination, the mature mRNAs are locally translated. mRNA localization coupled with local translation is thought to increase thermodynamic efficiency compared to protein trafficking and provides cells with a metabolic savings by localizing highly stable mRNAs distally and translating them only when needed (Holt and Bullock, 2009; Buxbaum et al., 2015; Fernandopulle et al., 2021). These combined processes also facilitate rapid localized responses to signaling cues, such as within synapses (Holt and Bullock, 2009; Buxbaum et al., 2015). Moreover, maintaining translationally quiescent mRNAs during the process of localization can prevent ectopic interactions between proteins, thus spatially limiting protein function or preventing cellular toxicity. For example, myriad neurodegenerative disorders are correlated with the inappropriate formation of nuclear or cytoplasmic ribonucleic protein particle (RNP) aggregates that are thought to be toxic to neurons (Abouward and Schiavo, 2021; Fernandopulle et al., 2021). Furthermore, localized translation could also be used to control the local assembly of large multi-protein complexes.

RNA localization is highly prevalent across eukaryotes and has also recently been described in prokaryotes (Engel et al., 2020; Irastortza-Olaziregi and Amster-Choder, 2021). The widespread nature of RNA localization is underscored by the fact that during *Drosophila* embryogenesis nearly 70% of transcribed mRNAs are asymmetrically localized (Lécuyer et al., 2007). While it has been long established that mRNAs such as *bicoid (bcd)*, *nanos (nos)*, and *oskar* (*osk*), are also asymmetrically localized during *Drosophila* oogenesis, multiple maternal mRNAs have also been recently shown to be localized and locally translated within murine oocytes (Jansova et al., 2021). Additionally, many studies have shown widespread use of RNA localization within the nervous system. For example, a significant proportion of neuronal mRNAs in the rat hippocampus are enriched in neurites, as compared to the soma (Cajigas et al., 2012). Extensive RNA localization is also seen within endfeet of radial glial cells, a neural progenitor cell of the developing mammalian cortex (Pilaz et al., 2016; D’Arcy and Silver, 2020). Importantly, transcriptomic analyses of cytoplasmic and nuclear RNAs from human fetal and adult brain tissue suggest that thousands of RNAs have subcellular localization during human neurogenesis (Price et al., 2020; Zaghlool et al., 2021). Moreover, the subcellular localization patterns change significantly from prenatal to adult stages, highlighting a potential role in RNA localization regulating neurogenesis. Notably, many of the genes that show differential localization within neurons are associated with neurological disease (Price et al., 2020). While there have been efforts to understand the mechanisms and functional relevance of RNA localization, few individual RNAs have been studied within this capacity, especially relative to the number of RNAs that are known to be localized within cells.

Finally, RNA localization has been demonstrated to have functional importance. Mislocalization of multiple maternal mRNAs, such as *nos, bcd*, and *osk* in the *Drosophila* oocyte has long been known to cause severe embryonic segmentation patterning defects (Driever and Nüsslein-Volhard, 1988; Ephrussi et al., 1991; Gavis and Lehmann, 1992). Similarly, mislocalization of *nanos* mRNA results in neuron dysmorphogenesis in the *Drosophila* larval peripheral nervous system (Brechbiel and Gavis, 2008). Many mRNAs have also been shown to be localized to centrosomes in diverse cell types and within many different organisms (Ryder and Lerit, 2018; Ryder et al., 2020). Significantly, mistargeting of the centrosomally localized mRNA *centrocortin* within the *Drosophila* embryo results in mitotic spindle defects, disrupted nuclear division and centrosome duplication defects (Ryder et al., 2020).

The observation that mRNA localization is prevalent, highly conserved, and with functional importance, highlights the need to understand the mechanisms that regulate mRNA localization. RNA localization is often achieved using active transport mechanisms (reviewed by Engel et al., 2020), diffusion and trapping, as well as localized degradation of mRNAs. More recently, the usage of alternative transcripts, via alternative splicing, has also emerged as a regulatory mechanism for RNA localization.

## Alternative Isoforms and Localization

**FIGURE 2:**
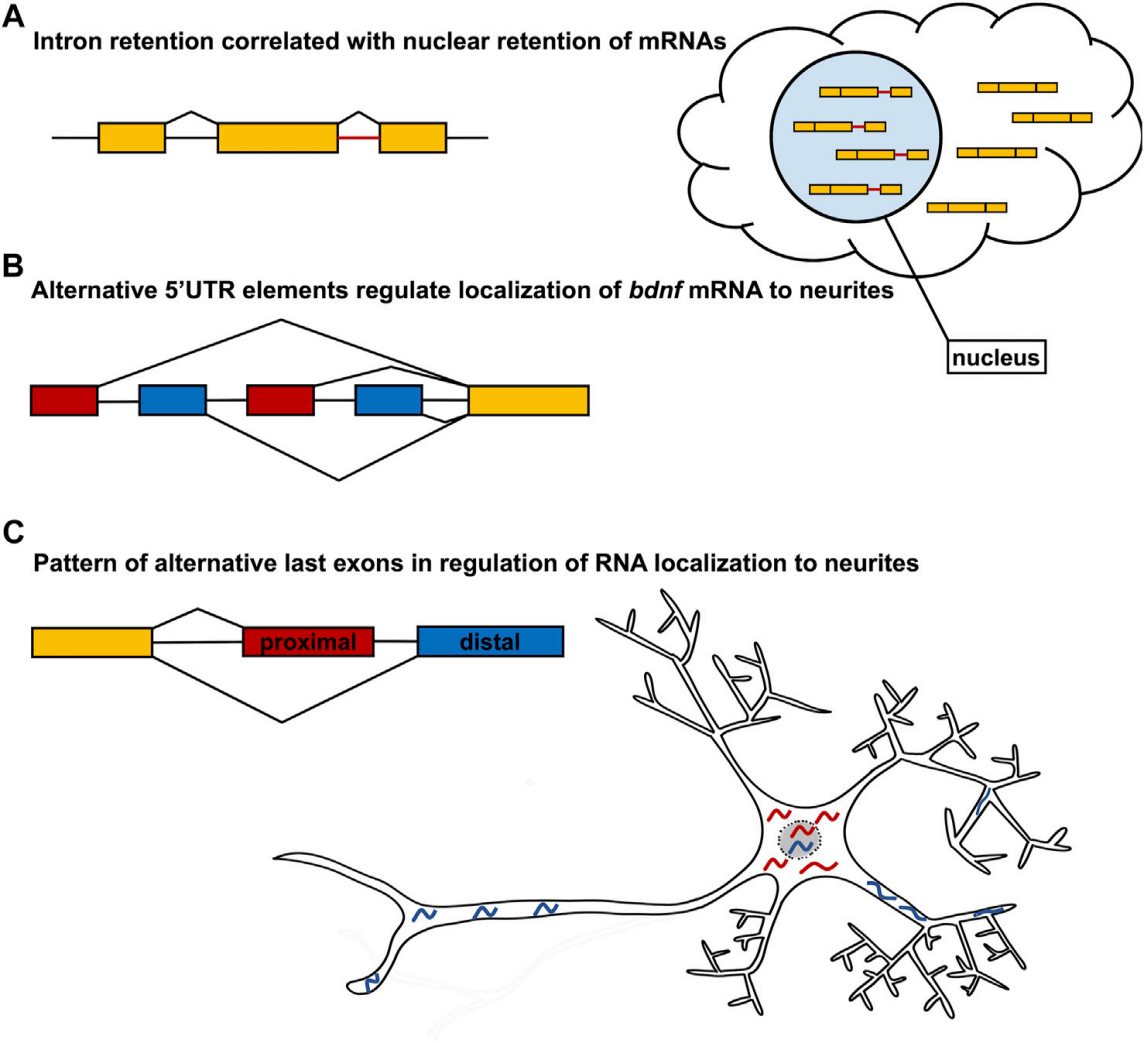
Alternative Splicing regulates mRNA localization. **(A)** Intron retention has been demonstrated to be one of the most abundant splicing events that regulates nuclear retention of mRNA. **(B)** Alternative first exons with distinct 5′UTR sequences regulate *bdnf* mRNA localization to neurites as demonstrated in the neuron model. The gene model presented does not represent all possible alternative first exons of *bdnf*, but just the relative positions of the transcripts analyzed in Baj et al. (2011). **(C)** Large scale analysis of neurite and soma enriched alternative transcripts identifies the usage of alternative last exons as being one of the largest contributors to differential localization. More specifically, the use of distal last exons more strongly correlates with neurite localization whereas the use of proximal last exons more strongly correlates with soma localization. An example of this is shown in the neuron model.

Alternative splicing has recently emerged as an important regulatory mechanism for mRNA localization. By selecting for or against exons containing specific localization elements, splicing machinery can precisely control the destination and timing of RNA localization. Alternative splicing can impact multiple stages of mRNA localization including both nuclear export and subcellular localization within the cytoplasm. In a recent survey of transcript variants in 13 human cell lines, over 8,000 genes were identified that contain transcript switches, or variant isoforms where one was preferentially localized to the nucleus and another was preferentially localized to the cytoplasm (Zeng and Hamada, 2020; Figure 2). Interestingly, the only splicing event that was significant for transcript switching from nucleus to cytoplasm was intron retention, which primarily signaled an mRNA to remain within the nucleus (Zeng and Hamada, 2020; Figure 2A). In yeast, two alternative splicing factors, G-strand binding protein (Gbp2) and Hypothetical RNA-binding protein 1 (Hrb1), were observed to preferentially bind intron-retaining mRNAs and serve as a switch between mRNA degradation and nuclear export (Hackmann et al., 2014). This suggests that the nuclear retention of intron-retaining mRNAs could serve as a process of quality control to ensure non-functional isoforms do not exit the nucleus to be translated. However, it is plausible that nuclear retention of mRNAs may serve other regulatory functions yet to be identified. Although some alternative splicing events may impact the nuclear-cytoplasmic shuttling of mRNA, many alternative splicing events have been demonstrated to regulate subcellular localization. For example, in rat hippocampal neurons, the localization of *bdnf* mRNA was demonstrated to be regulated by alternate 5′UTR elements (Baj et al., 2011; Figure 2). All rat *bdnf* transcript variants contain a common coding region. However, 22 different spliceforms are generated through alternative splicing of different 5′UTR containing exons and alternative polyadenylation sites within the 3′UTR (Baj et al., 2011; Figure 2B). The visualization of spliceforms with one of four different 5′UTR exons showed that transcripts containing exons 1 and 4 localize within 40 μm of the soma. However, variants containing exons 2C and 6 localize to the distal neurites up to 70 μm from the soma (Baj et al., 2011; Figure 2B). Although the localization of *bdnf* mRNA is regulated by elements in the 5′UTR, it has also been shown that differences in 3′UTR sequence also regulate *bdnf* mRNA localization within neurons (An et al., 2008). In particular, different polyadenylation sites within the *bdnf* 3′UTR create a short (350 bp) transcript and a long (2,850 bp) isoform, of which the long isoform is targeted to dendrites of hippocampal neurons. Loss of the long 3′UTR *bdnf* transcript results in dendritic morphology defects, as well as physiological consequences. Indeed, regulation of mRNA localization is most consistently imparted by elements in the 3′UTR (Gavis and Lehmann, 1992; Ferrandon et al., 1994; Kislauskis et al., 1994; Mayford et al., 1996; Chang et al., 2004; Gore et al., 2005; Jain and Gavis, 2008; Racca et al., 2010; Perry et al., 2012; Taliaferro et al., 2016; Ciolli Mattioli et al., 2019; Bae and Miura, 2020). For example, the fusion of the *nos* 3′UTR to a lacZ translational reporter recapitulates endogenous *nos* mRNA localization in *Drosophila* embryos (Gavis and Lehmann, 1992) Moreover, the 3′UTR of *nos* is required to localize the *nos* transcript to neurites of sensory neurons in *Drosophila* larvae (Brechbiel and Gavis, 2008). Furthermore, fusion of the *bcd* 3′UTR to the *nos* RNA is sufficient to localize *nos* RNA to the anterior pole of the *Drosophila* embryo, in a pattern indistinguishable from the endogenous localization of *bcd* mRNA (Gavis and Lehmann, 1992). More recently, it has been shown that specific 3′UTR sequences are utilized to localize transcripts in distinct patterns within the myelin sheath of the developing zebrafish nervous system. And although different UTR sequences can dictate distinct localization patterns within myelin, some RNAs encoded by different genes share common localization sequences that are sufficient to localize reporter constructs to the myelin sheath (Yergert et al., 2021). Finally, disruption of Pumilio-binding sites in the 3′UTRs of RNAs that encode many distinct chemotaxis pathway components, results in disrupted cell migration in the social amoeba *Dictyostelium,* further supporting widespread use of 3′UTR elements in RNA localization (Hotz and Nelson, 2017).

One of the more recent developments in localization is the impact of alternative last exons (ALEs), which can generate distinct 3′UTR sequences in different spliceforms, on the localization of mRNA isoforms. In N2A and CAD mouse neuronal cell cultures, over 800 genes were identified that had isoforms differentially enriched in the neurites or the soma. Interestingly, of the isoforms analyzed, the highest frequency splicing event of differentially localized isoforms was found to be alternative last exons and tandem 3′UTRs, as compared to the alternative first exon or skipped exon splicing events. In 80% of the isoform pairs with ALEs, the selection of the distal ALE coincided with localization to the neurites and selection of proximal ALEs to the soma (Figure 2C). Furthermore, when reporter genes were fused with the differentially localized ALEs, those containing distal ALEs localized to neurites, however those containing proximal ALEs had reduced localization to neurites. This suggests that the 3′UTR elements of the ALEs are sufficient to guide subcellular localization within neurons (Figure 2). Moreover, the bias for neurite-localized transcripts to utilize distal ALEs was also observed during the differentiation of human neural precursor cells, suggesting that the mechanism is conserved across species. Furthermore, many of the genes containing neurite-localized distal ALEs encode neurotransmitter receptors, ion channels, and trafficking proteins, suggesting that the preferential expression of distal ALEs ensures local translation of molecules necessary for proper functioning of mature neurons (Taliaferro et al., 2016). Interestingly, this trend is reversed during the reprogramming of human fibroblasts to induced pluripotent stem cells (iPSCs) and in various cancers, where there is preferential expression of proximal ALEs. This suggests that the usage of proximal ALEs may be inversely associated with cell differentiation. Furthermore, cleavage and polyadenylation (CPA) factors were identified as playing a potential role in the selection of the ALEs and tandem 3′UTRs. The depletion of CPA factor CFIM25/cleavage and polyadenylation specificity factor subunit 5 (Cpsf5) resulted in differential expression of proximal ALEs, whereas the depletion of the CPA factor cleavage stimulation factor subunit 2 tau variant (CSTF2T) resulted in differential expression of distal ALEs (Taliaferro et al., 2016). This would suggest that fine-tuned control of these CPA factors and other RBPs may regulate the shift from proximal ALE selection to distal ALE selection during neuron differentiation.

Another recent study utilized mouse embryonic stem cells (mESCs) to identify RNA isoforms that differentially localize between the soma and neurites. 593 different genes were found to have RNA isoforms with distinct 3′UTRs, generated either from ALE usage or alternative polyadenylation, that differentially localized to the soma versus neurites. Furthermore, the distinct 3′UTRs for cell division cycle 42 (*CDC42*) mRNAs were sufficient to localize reporter constructs to their respective compartments. Interestingly, there was also a bias for shorter 3′UTRs to be localized to neurites as compared to the soma (Ciolli Mattioli et al., 2019). However, examples of long 3′UTR isoforms conferring dendritic localization have also been reported (An et al., 2008). Taken together, alternative splicing of noncoding exons may not necessarily affect protein sequence but may have very real functional consequences by regulating the subcellular localization of specific RNA transcripts.

## Functional Isoforms

Thus far, we have focused on how alternative splicing can regulate RNA localization and stability. However, alternative splicing can also produce vastly different protein isoforms for a given gene. The difference in protein sequence between isoforms suggests that the same gene can produce proteins with different and sometimes opposing functions. Indeed, there is evidence that different isoforms can have variable impacts on different developmental pathways. However, the ability to elucidate the different functions of distinct protein isoforms has proven difficult. For one, proteomic analysis has only been able to detect a small number of alternative protein isoforms, even when transcripts are present (Tress et al., 2017). This could be the result of the low abundance of alternative isoforms, especially since there is a high degree of tissue specificity in the expression of alternative protein isoforms (Morris et al., 2012; Gu et al., 2019; Rodriguez et al., 2020). Alternatively there could be mechanisms that reduce the rate of translation of certain alternative protein isoforms (Beerman and Jongens, 2011). Finally, in genes with more than one alternative protein isoform, few genetic tools, such as isoform-specific mutations, have been generated to properly elucidate the isoform-specific functions (Gu et al., 2019). Despite these challenges, several studies have been able to elucidate isoform-specific functions for select genes.

For example, the mouse *kinesin family member 2A* (*Kif2a*) gene, a microtubule depolymerizing kinesin, has three different protein isoforms. The isoforms are differentiated by the inclusion/exclusion of exon 18, encoding 37 amino acids, or the alternative 5′ site selection of exon 5, which alters the peptide sequence by 20 amino acids (Akkaya et al., 2021). *Kif2a* is known to be important for dendrite development and radial neuron migration. When coupling *Kif2a* shRNA knockdown with the inclusion of shRNA resistant cDNA of specific isoforms, all isoforms rescue aberrant dendritic morphology in Neuro2a cell culture. However, only the isoforms containing the long version of exon 5, KIF2A.1 and KIF2A.2, rescue radial migration defects of cortical neurons. Immunofluorescence imaging shows no significant difference in the localization of isoforms, suggesting any functional differences can be attributed to variations in protein sequence (Akkaya et al., 2021). Furthermore, proximity-labeling was used to identify isoform specific interactors. While KIF2A.1 and KIF2A.2 interactors were enriched with mitochondrial proteins, interactors of the isoform containing the short form of exon 5, KIF2A.3 were not. Furthermore, KIF2A.1 and KIF2A.2, but not KIF2A.3, co-localize with mitochondria, further supporting mitochondrial related functions for only a subset of the KIF2A protein isoforms (Akkaya et al., 2021).

There is also evidence to suggest that alternative splicing that results in the production of distinct protein isoforms is tissue specific. In one study that examined 255 alternative splice events, nearly 40% of the splicing events resulted in tissue-specific expression of an alternative protein isoform (Rodriguez et al., 2020). This tissue specificity is highlighted by the *Drosophila Collapsin Response Mediator Protein (CRMP)* gene. Immunofluorescence imaging of larvae with GFP-tagged protein isoforms that mutually exclude the amino acid sequences encoded by either exon 9a or 9b reveals that isoforms containing the protein sequence encoded by exon 9a are exclusively in non-neuronal tissue and those containing the protein sequence encoded by exon 9b are exclusively in neuronal tissue (Morris et al., 2012). Furthermore, the two different protein isoforms of the *CRMP* gene in *Drosophila* are orthologous to two entirely different genes in humans. The spliceform containing exon 9a is orthologous to human *dihydropyrimidase (DPYS)* and contains a conserved histidinyl residue important for pyrimidine catabolism. Conversely, the isoform containing exon 9b is orthologous to human *CRMP1*. Additionally, using an assay to identify pyrimidine catabolism through the suppression of black pigmentation in *black* mutant animals, only flies with a functional isoform orthologous to human *DPYS* demonstrated functional pyrimidine catabolism. By contrast, flies with only a functional isoform orthologous to human *CRMP1* did not appear to have functional pyrimidine catabolism (Morris et al., 2012).

The *Transient receptor potential cation channel A1 (Trp1A)* gene in *Drosophila* is another gene with multiple protein isoforms, produced through alternative splicing, that have distinct functions. *Trp1A* is an ion channel that detects noxious stimuli including heat, reactive oxygen species (ROS), UV light, and irritant chemicals (Gu et al., 2019). *Trp1A* is expressed in the larval midgut, corpus cardiacum cells, and within the nervous system. However, although there is some overlap in expression, the 5 alternative protein isoforms have distinct expression patterns from another (Gu et al., 2019). The variant isoforms are produced from the splicing of an alternative first exon and/or the splicing of mutually exclusive exons 12 and 13. Tissue specific knockdown of *Trp1A* using the Gal4-UAS system shows that *Trp1A* is necessary in Class IV dendritic arborization (C4da) neurons for nociceptive response to heat. The local administration of heat to larvae can stimulate a rolling behavior in what is termed a heat avoidance assay. This rolling behavior is lost when *Trp1A* is knocked down in C4da neurons. Interestingly, only 3 of the isoforms (C, D, and E) are expressed in C4da neurons (Gu et al., 2019). To examine the role of isoform specific expression in nociceptive behavior, transgenic lines that contained knock-ins of specific TRP1A protein isoforms in a *Trp1A* knock out background were developed and assessed using the heat avoidance assay. Only the knock-in of isoform D was able to rescue the nociceptive response in larvae, highlighting isoform specific functions of TRP1A in nociception (Gu et al., 2019). Additionally, Ca^2+^ imaging showed C4da neuron activation in response to noxious heat only when isoform D was knocked in. Furthermore, an isoform specific transgenic line that simultaneously knocked out isoform A and D resulted in abolished rolling behavior, whereas knockout lines of the other isoforms were indistinguishable from wildtype (Gu et al., 2019). Similarly, isoform D specifically mediates the nociceptive response to the chemical irritant allyl isothiocyanate (AITC). However, the nociceptive response to the production of H_2_O_2_ induced by UVC exposure, is only mediated by the knock-in of isoform C in the *Trp1a* knockout background, as assessed through the rescue of rolling behavior (Gu et al., 2019). Thus, the two different TRP1A protein isoforms, C and D, both play a role in nociceptive response, but they are sensitive to different types of stimuli. However, it is important to note that overexpression of either C or D in a *Trp1a* knockout line was sufficient to rescue rolling behavior to all stimuli (Gu et al., 2019). This suggests that both isoforms are capable of response to all the stimuli but may have varying degrees of sensitivity or a different threshold. Furthermore, overexpression of isoforms A, B, C, or D in a wildtype background resulted in heat allodynia, or the lowering of the heat threshold for nociceptive response suggesting all four isoforms may play a role in nociception. Interestingly, only isoform E did not have any observable function in nociception. This could suggest that isoform E is a non-functional byproduct of alternative splicing, or alternatively, that the function of isoform E has yet to be identified (Gu et al., 2019). Overall, the identification of tissue specific expression of protein isoforms coupled with functional differences among isoforms suggests that alternative splicing can be used to regulate protein structure to ensure protein functions that are tailored to a tissue’s unique and specific needs.

In *Drosophila*, the transcription factor *longitudinals lacking* (*lola*), has been shown to have 19 protein isoforms, all of which include four 5′ exons that encode the N-terminal BTB dimerization domain coupled with an alternatively spliced fifth exon (Goeke et al., 2003). 17 of the 19 isoforms also contain either a pair of zinc-finger domains or a single zinc-finger domain. Interestingly, alignment of the zinc-finger domains suggests that each of these 17 variants has different DNA contact residues. This implies that each variant could have distinct transcriptional targets (Goeke et al., 2003). Furthermore, RT-PCR revealed that distinct combinations of these spliceforms are expressed across various life stages and tissues. Finally, although dysfunction of *lola* has been demonstrated to cause CNS specific phenotypes and to also affect the development of the ISN_b_ peripheral nerve, mutations specific to either the K or L isoforms impact development of the ISN_b_ nerve with only mild effects on CNS function (Goeke et al., 2003). Taken together, this suggests while all Lola protein isoforms may function in transcriptional regulation, specific isoforms may regulate distinct targets during different developmental stages and tissues.

“Exon-activated functional rescue” (EXAR), in which specific alternative isoforms can rescue the activity of another gene, was recently described for the RBP, Found in Neurons (FNE), during *Drosophila* embryogenesis (Carrasco et al., 2020). The RBP encoded by *embryonic lethal abnormal vision (elav*) is important for the neuronal specific alternative cleavage and polyadenylation (APA) of multiple RNA targets (Carrasco et al., 2020). Interestingly, another protein of the ELAV-family, FNE, can compensate for certain ELAV functions. For example, neuronal APA is abolished in *Δfne Δelav* double mutants, but neuronal APA is not impacted in *Δfne* single mutants (Carrasco et al., 2020). Furthermore, in *elav* mutant animals, *fne* is alternatively spliced to include a mini-exon that is not expressed in wild-type *Drosophila* (Carrasco et al., 2020). The inclusion of the mini-exon increases the amino acid sequence similarity of ELAV and FNE between the second and third RNA recognition motifs, and facilitates nuclear localization of FNE, which is not observed in wildtype *Drosophila.* These changes confer the ability of FNE to carry out neuronal APA and therefore rescue ELAV function (Carrasco et al., 2020). Indeed, deletion of the mini-exon is sufficient to abolish nuclear localization of FNE in an *elav* mutant background and reduced the FNE-mediated rescue of neuronal APA (Carrasco et al., 2020). This data suggests that the presence of ELAV represses the inclusion of the *fne* mini-exon. However, in the absence of ELAV, the mini-exon is included resulting in the nuclear localization of FNE and the rescue of neuronal APA (Carrasco et al., 2020).

However, it is important to note that not all alternative protein isoforms result in differing functions. For example, *Drosophila Fmr1* encodes two major isoforms that have ∼7 kDa size difference that is generated by the use of a non-canonical CTG alternative translation site upstream of the canonical ATG start codon (Beerman and Jongens, 2011). dFMR1 is necessary for proper motor neuron development and axon guidance in mushroom bodies; *fmr1* dysfunction results in an increased number of boutons and aberrant crossing of the β-lobe of the mushroom body through the midline of the brain (Beerman and Jongens, 2011). Interestingly, only the expression of the small isoform is capable of rescuing both phenotypes, whereas the expression of the large isoform is only capable of partial rescue. However, the expression level of the large isoform is significantly lower than the small isoform, likely due to the non-canonical start codon. Thus, when the large isoform was overexpressed by changing the non-canonical start codon to a canonical ATG start codon, the neural phenotypes were rescued. This suggests that the two isoforms share the same function, and that any aberrant phenotypes seen in the isolated expression of one isoform can be attributed to expression levels and not functional differences (Beerman and Jongens, 2011). However, it also plausible that the two isoforms have differing functions that have yet to be identified. Indeed, *Fmr1* has myriad developmental roles that have not been investigated in an isoform specific manner.

## Conclusion

It has long been recognized that the use of alternative splicing can generate different transcript isoforms from a single gene. Transcriptomic analyses have also begun to shed light on the tissue-specific, sex-specific and age-specific splicing patterns in various organisms. Future work will determine the extent to which these splicing patterns are conserved. Nonetheless, much remains to be learned with respect to the functions of different spliceforms. Historically, researchers have used the term “functional splicing” to describe the alternative splicing of RNAs to create distinct protein products. By contrast, isoforms that do not change the protein sequence and may instead be regulatory are referred to as “unproductive splicing” events. However, we suggest that this protein-centric terminology is rather misleading. Indeed, while alternative splicing of noncoding exons may not change the protein product of an mRNA spliceform, it can most certainly provide an important regulatory function. In fact, we have only just begun to appreciate the prominent impact of PE regulation in development and disease. Yet the recent link between aberrant PE regulation in cancer and neurological disorders provides promising avenues in developing novel therapeutic strategies. Thus, further research on the functional roles of PEs will increase our understanding of gene regulation and the genetic mechanisms underlying disease. Similarly, the observation that ALEs are differentially used in cancer and cell differentiation suggests that splicing of ALEs serves an important role within the cell and can ultimately affect protein function by regulating transcript localization. Thus, we propose instead to use the term “regulatory splicing”, as opposed to “unproductive” or “nonfunctional” splicing, to refer to the alternative splicing of noncoding exons that may affect the subcellular localization or stability of an RNA transcript. Moreover, a change in terminology should provide more accuracy and garner a greater awareness of the possible different functions that distinct spliceforms may have in the cell.

## Supplementary Material

Supplementary Table S1 List of NMD-sensitive or PTC-containing genes across Metazoa.
